# Exogenous γ-aminobutyric acid application delays onset of ripening in Grenache, Chardonnay, and Shiraz grape berries

**DOI:** 10.1093/pcp/pcag078

**Published:** 2026-06-22

**Authors:** Chethana G Shekharappa, Nicholas J Booth, Vinay Pagay, Stephen D Tyerman, Sunita A Ramesh

**Affiliations:** School of Agriculture, Food and Wine, Waite Research Institute, Adelaide University, PMB1, Glen Osmond, SA 5064, Australia; Transport Physiology and Signaling Lab, Biological Sciences, College of Science and Engineering, Flinders University, Bedford Park, SA 5042, Australia; School of Agriculture, Food and Wine, Waite Research Institute, Adelaide University, PMB1, Glen Osmond, SA 5064, Australia; School of Agriculture, Food and Wine, Waite Research Institute, Adelaide University, PMB1, Glen Osmond, SA 5064, Australia; School of Agriculture, Food and Wine, Waite Research Institute, Adelaide University, PMB1, Glen Osmond, SA 5064, Australia; Transport Physiology and Signaling Lab, Biological Sciences, College of Science and Engineering, Flinders University, Bedford Park, SA 5042, Australia

**Keywords:** living tissue percentage, berries, delay, γ-aminobutyric acid (GABA), ripening, *Vitis vinifera*

## Abstract

Physiological and biochemical changes in *Vitis vinifera L*. (grape) berries during development have been extensively studied; however, the role of γ-aminobutyric acid (GABA), a non-protein amino acid that changes during berry development, remains unknown. We hypothesized that GABA accumulation in the berry may have an osmoprotective role and increase the living tissue percentage during development. On this precedent, exogenous application of GABA (5 mM) to bunches may result in a delayed onset of ripening. GABA was observed to accumulate concomitantly with sugar accumulation commencing at veraison in Chardonnay, Shiraz, and Grenache grape berries sampled from the Coombe vineyard over two seasons (2018–20). Cultivar-dependent variability was noted in hydrogen peroxide and antioxidant capacity. In berries treated with exogenous GABA (5 mM) weekly from the pea-stage until veraison, decreases in berry mass, total soluble solids, and cell death, along with increased antioxidant capacity, and a delay in veraison were observed. These findings suggest that exogenous GABA may modulate ripening, although the exact mechanisms require further investigation. Elucidating these mechanisms may benefit growers by improving grape berry quality and enabling modification of ripening in response to a changing climate or adverse weather events.

## Introduction

Viticulture is particularly sensitive to climatic variability because high-quality berries are essential for a balanced wine, requiring the appropriate accumulation of sugars, acids, aromas, and flavour compounds (Jones et al. 2005). Increasingly frequent extreme heat and drought events can trigger premature veraison, disrupt berry development, and generate unfavourable metabolic profiles, particularly in warm regions, such as McLaren Vale and Roussillon (Mullins et al. 1992, Jones et al. 2005). These conditions reduce vine productivity and berry quality through sunburn, shrivel, altered sugar concentrations, and imbalanced alcohol levels (Sharma et al. 2012), highlighting the need for strategies that delay ripening and shift the harvest outside of hot, dry periods. Ripening from veraison involves softening, anthocyanin accumulation, declining acidity, increasing sugars, and progressive mesocarp cell death (Krasnow et al. 2008, Tilbrook and Tyerman 2008, Fuentes et al. 2010, Coetzee and du Toit 2012). Additional developmental changes include increased reactive oxygen species (ROS) at veraison (Pilati et al. 2014), the accumulation of γ-aminobutyric acid (GABA) (Pereira et al. 2006, Ali et al. 2011), and shifts in antioxidant activity.

GABA, a non-protein amino acid, has multiple roles in plant growth and development, including the maintenance of carbon/nitrogen balance, osmotic adjustment, pH regulation, ROS regulation, and stress signalling (Bown and Shelp 1997, Bouché and Fromm 2004, Shelp et al. 2012, Michaeli and Fromm 2015, Ramesh et al. 2015, Ramesh et al. 2017, Yuan et al. 2023). In Merlot berries, shaded fruit accumulates more GABA in skin and pulp than light-exposed berries (Pereira et al. 2006). Metabolic profiling of Trincadeira, Touriga Nacional, and Argones using Nuclear Magnetic Resonance (NMR) revealed cultivar-dependent differences in phenolics, organic acids, sugars, and amino acids, including GABA, with higher GABA at ripening and harvest and particularly elevated levels in Touriga Nacional alongside alanine, valine, and glutamate (Ali et al. 2011). Under drought stress, Meski leaves accumulate more proline, arginine, glutamate, and GABA than Chardonnay (Hatmi et al. 2015), and nitrogen fertilization increases GABA in White Riesling leaves between flowering and fruit set (Schaller 2013). Although physiological and biochemical changes during berry development are well characterized, the role of GABA in berry development remains unclear. GABA accumulation may protect berries from hydrogen peroxide (H₂O₂)-induced oxidative damage, but the mechanisms involved are unknown.

Current approaches to improve grape quality and shift the initiation of ripening include the preveraison application of naphthalene acetic acid and the auxin-like compound benzothiazole-2-oxyacetic acid in Shiraz and Merlot (Davies et al. 1997, Böttcher et al. 2011, Previtali et al. 2022b). Although exogenous GABA application enhances antioxidant capacity and reduces oxidative damage through ROS scavenging in many crops and in pre- and post-harvest fruits (Wang et al. 2014, Li et al. 2016, Li et al. 2019, Salah et al. 2019, Badiche-El Hilali et al. 2023, Feng et al. 2023), no study has examined its effects in grape berries. The hypotheses and objectives of this study were: (i) concentrations of GABA, H₂O₂ (ROS) and antioxidant systems [catalase (CAT) and ascorbic peroxidase (APX) enzymes] differ during development in Chardonnay, Shiraz, and Grenache berries; (ii) exogenous GABA application to berry bunches may delay the onset of ripening; and (iii) GABA has an osmoprotective role and increases the living tissue percentage (LT%) during development.

The three cultivars selected for the study differ in their degree of pericarp cell death during ripening. Berries from field-grown, own-rooted Grenache (not prone to shrivel), Shiraz (prone to shrivel and cell death), and Chardonnay (not prone to shrivel but shows cell death) were sampled from veraison [70–80 days after flowering (DAF)] over two seasons (2018–19 and 2019–20), and ripening-associated traits, including fresh berry mass, total soluble solids (TSS), H₂O₂, GABA, CAT, APX, and ethanol (EtOH), were measured. To assess whether exogenous GABA delays ripening, berries were also sampled from the pea-stage (35 DAF) to veraison from bunches treated weekly with GABA.

## Results

### Developmental changes between Chardonnay, Shiraz, and Grenache berry ripening

For both seasons 1 and 2, January was the warmest month, with the highest mean temperature, *T*_max_, vapour pressure deficit (VPD), and global solar radiation (GSR) (Table 1). In general, the climate during season 1 was more severe than in season 2, particularly later in the growing season during February and March, where all measured parameters were greater. The physical parameters measured varied between the three grape cultivars over the two seasons (Fig. 1). During season 1, all cultivars increased in biomass from veraison to harvest, and then a sudden decrease in berry mass was observed postharvest for both Chardonnay and Shiraz, but not Grenache. For all measured sampling points, Grenache berries had the greatest mass, followed by Shiraz and then Chardonnay. The interactions of developmental stage and cultivar on berry mass were extremely significant (*P* = 0.0009 and *P* < 0.0001, respectively) across both seasons (Fig. 1a and b).

**Table 1:** Environmental conditions during different phenological stages in 2018/2019 and 2019/2020 growing seasons.

2018/2019					2019/2020			
	Mean temperature (°C)	*T*_max_ (°C)	VPD (kPa)	Mean GSR (W/m^2^ )	Mean temperature (°C)	*T*_max_ (°C)	VPD (kPa)	Mean GSR (W/m^2^ )
**January**	31.9	41.8	2.71	825.8	31.3	43.5	1.19	941.2
**February**	29.2	38.7	2.16	805.1	27.1	33.6	0.62	770.1
**March**	26.5	40.4	1.55	632.3	26.1	33.2	0.60	228.4

**Figure 1:**
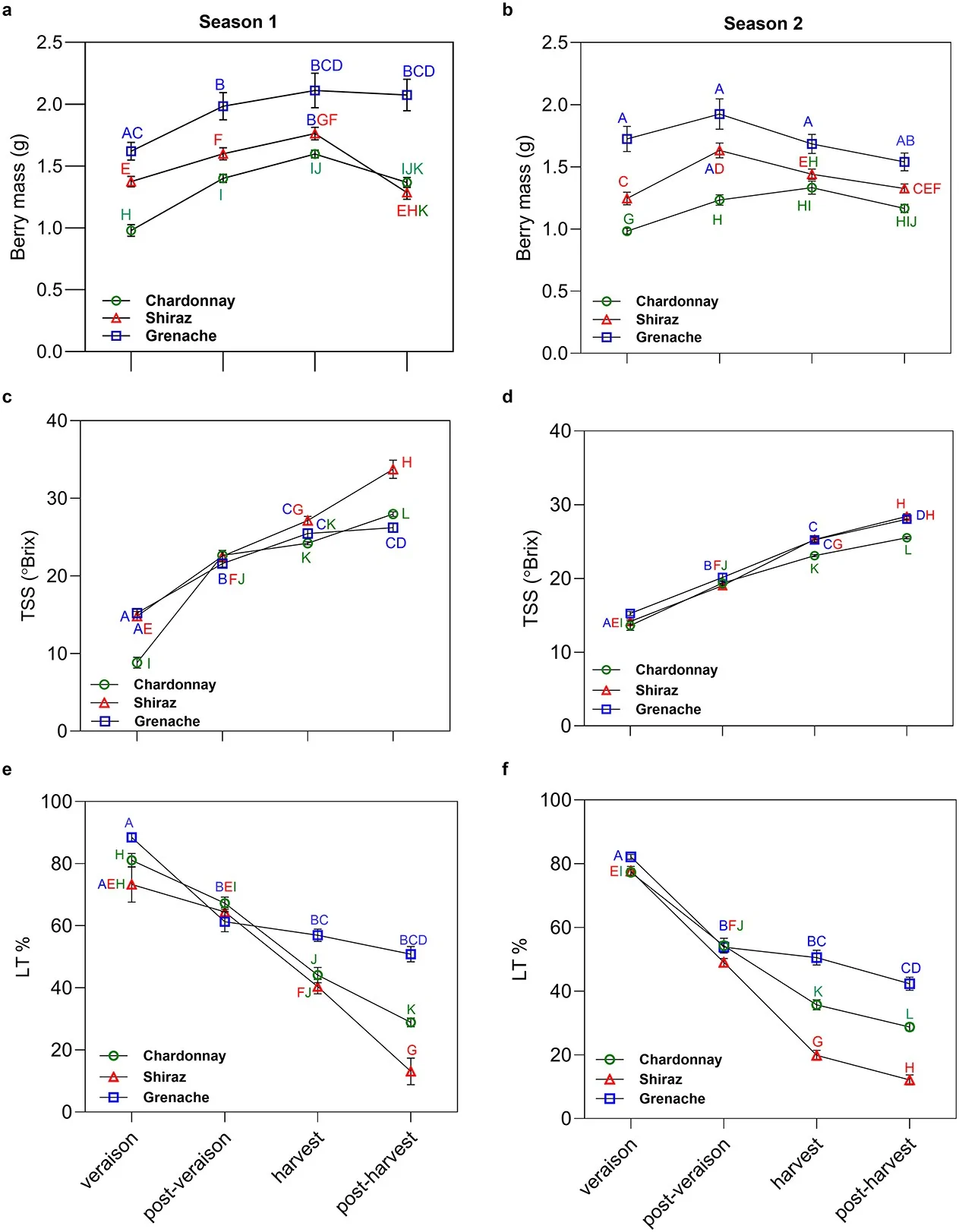
Physical changes of Chardonnay (veraison season 1: 05 January 2019 and season 2: 11 January 2020; circle), Shiraz (veraison season 1: 12 January 2019 and season 2: 12 January 2020; triangle), and Grenache (veraison season 1: 19 January 2020 and season 2: 16 January 2020; square) berry development during seasons 1 and 2. Data for berry mass (a, b), TSS (c, d), and LT% (e, f) are means ± SEM, *n* = 4. Uppercase letters describe significance within a cultivar over development while lowercase letters describe significance between cultivars at a singular developmental stage according to Tukeys’ multiple comparison (two-way ANOVA repeated measures, *P* < 0.05).

TSS increased for all three grape cultivars as the berries ripened in both seasons 1 and 2, but by postharvest in both seasons TSS was greatest in Shiraz, followed by Chardonnay and Grenache (Fig. 1c or d). For both seasons, the stage of development interaction with TSS was significant (*P* < 0.0001). In season 1, these differences were more distinct, with Shiraz berries having the highest TSS (33.7 °Brix) at postharvest (120 DAF) compared to Chardonnay (27.9 °Brix) and Grenache (26.2 °Brix), while in season 2, both Shiraz and Grenache had similar TSS (28.0 °Brix), while Chardonnay was lower (25.5 °Brix). The differences in TSS between the Shiraz/Grenache and Chardonnay cultivars were significantly different postharvest for both seasons 1 and 2 (Fig. 1c or d). In both seasons, the percentage of living tissue in the grape pericarp (%LT) decreased with time, at veraison this was between 73% and 88%, but it was more variable at postharvest, with up to 50% LT for Grenache and as low as 12% LT for Shiraz (Fig. 1e or f). Shiraz and Chardonnay showed a steep negative linear trend in LT% across both seasons from veraison until harvest, but only a moderate decrease from harvest to postharvest. Whereas in Grenache, there was a decrease in LT% from veraison (80 DAF) to postveraison (100 DAF), but a decline in LT% from postveraison to postharvest (Fig. 2c and f). This corresponds to a significantly higher LT% in Grenache at postharvest when compared to Chardonnay and Shiraz (Fig. 1e and f; Fig. 2a).

**Figure 2:**
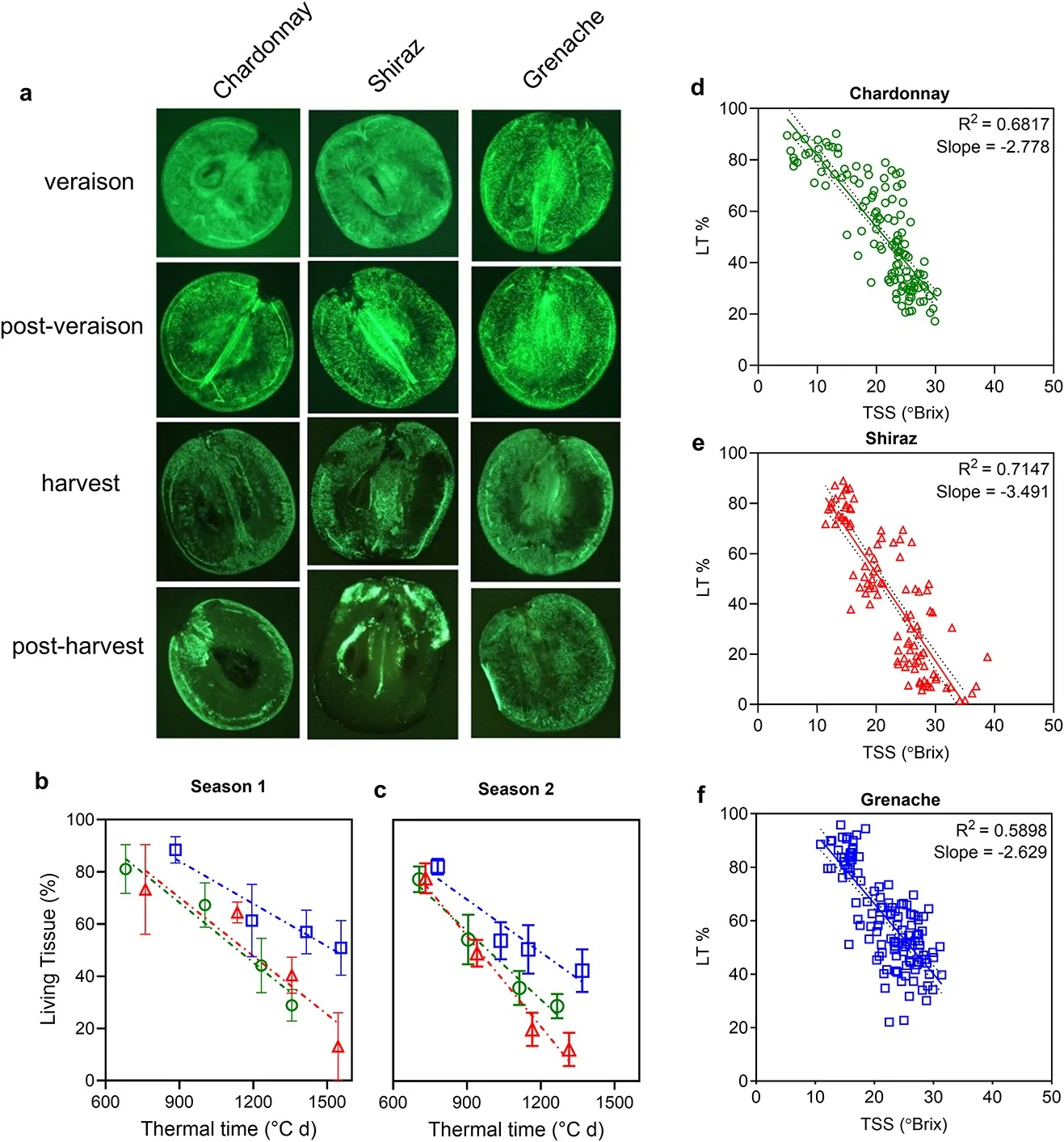
Living tissue decreases with developmental stage and increasing TSS. (a) Representative medial longitudinal berry sections stained with FDA highlighting LT% at different stages of ripening (corresponding to DAF). Green fluorescence indicates living tissue while black region indicates cell death. (b, c) Berry cell vitality as a function of thermal time; Chardonnay (green circle), Shiraz (red triangle), and Grenache (blue square); (d–f) regression analysis of the relationship between LT% and TSS within individual berries of Chardonnay (green circle), Shiraz (red triangle), and Grenache (blue square) and in seasons 1 and 2. Each data point is a single berry from four biological replicates in season 1 (2018–19) and season 2 (2019–20) combined.

A regression analysis found that TSS strongly correlates with LT% for all cultivars, with the strength of the correlation increasing with a decrease in LT% (*R*^2^ Chardonnay = 0.682; Shiraz = 0.715; Grenache = 0.590) (Fig. 2b–d). The slope of this relationship was greatest for Shiraz (−3.49 LT%/°Brix), but similar between Chardonnay (−2.78 LT%/°Brix) and Grenache (−2.63 LT%/°Brix), suggesting that as development proceeds, an increase in TSS is accompanied by lower LT% in all cultivars.

### Chardonnay, Shiraz and Grenache differ in their biochemical response to ripening

At veraison, EtOH concentrations ranged between 0.014 and 0.033 mg mL^−1^ but increased with berry maturation in all the three cultivars. This accumulation was significant in all cultivars (*P* = 0.03) and across cultivars (*P* = 0.0079) (Fig. 3a and b). This separation between the cultivars was most pronounced at the time of harvest but less pronounced in season 2. For both seasons 1 and 2, the H_2_O_2_ concentrations in Chardonnay and Shiraz increased throughout development whereas in Grenache, the concentrations initially spiked from the pea-stage to veraison, followed by a gradual decrease until postharvest (Fig. 3c and d). The interactions observed between stages of development across a cultivar and between cultivars for H_2_O_2_ was extremely significant (*P* < 0.0001; Fig. 3c and d) across both seasons. The H_2_O_2_ concentrations postharvest were the highest in Shiraz [21.9 and 18.5 nmoles/g fresh weight (FW)] among all three cultivars in both seasons, but at the veraison stage the greatest concentration of H_2_O_2_ was observed in Grenache in both seasons 1 (18.5 nmoles/g FW) and 2 (20.2 nmoles/g FW).

**Figure 3:**
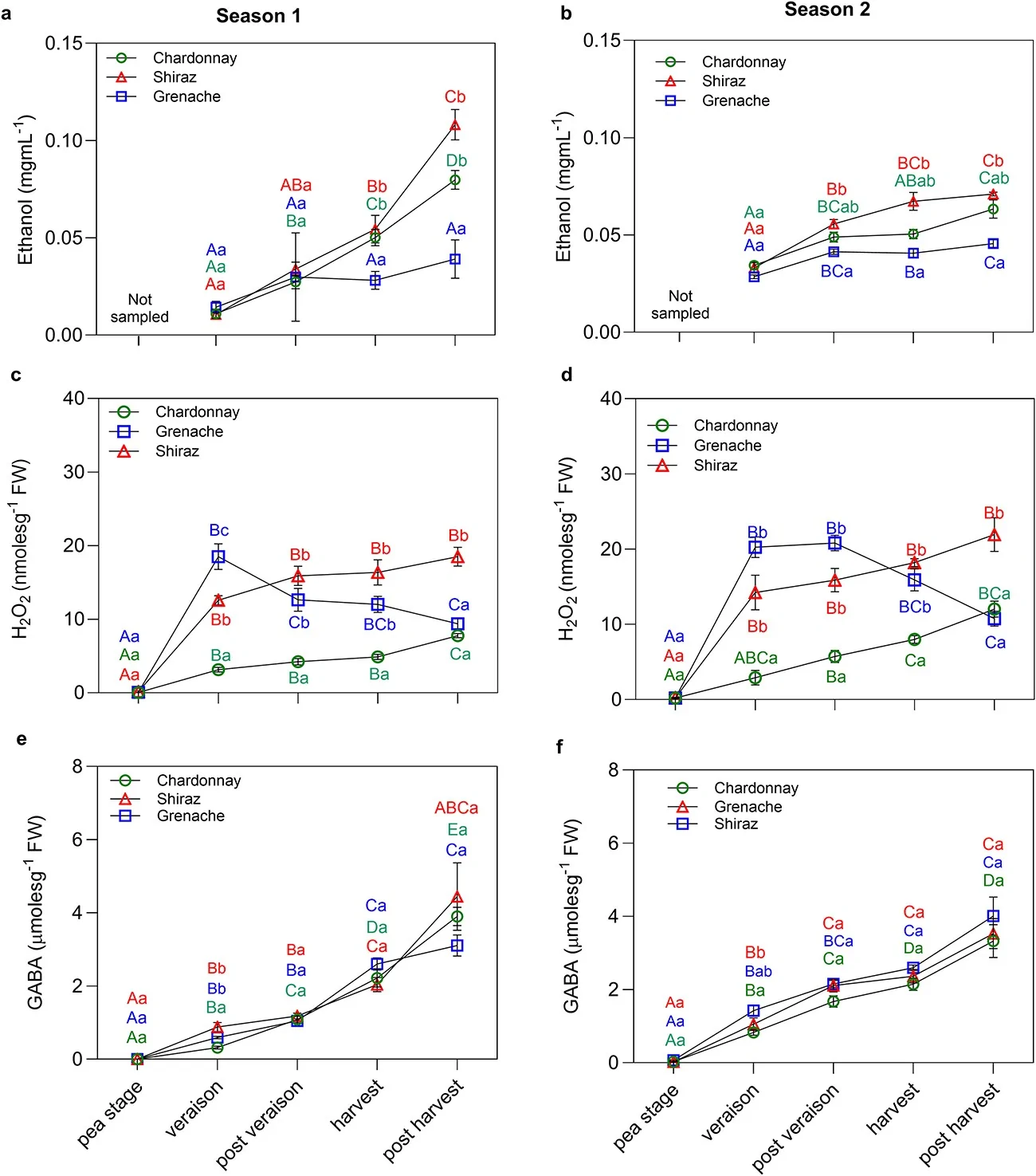
Changes in concentrations of endogenous GABA, H_2_O_2_, and EtOH in berries of Chardonnay (green circle), Shiraz (red triangle), and Grenache (blue square) berry development during seasons 1 and 2. Data for FW (a, b), TSS (c, d), and LT% (e, f) are represented as ±SEM, *n* = 4. Uppercase letters describe significance within a cultivar across development while lowercase letters describe significance between cultivars at a singular developmental stage according to Tukeys’ multiple comparison (two-way ANOVA repeated measures, *P* < 0.05).

For all three cultivars, endogenous GABA levels increased with berry development (Fig. 3e and f). Although significant differences in GABA concentrations could be determined across developmental stages (*P* < 0.0001), there were minimal varietal differences observed at veraison for Chardonnay and Shiraz (Fig. 3e and f). Weak negative correlations between H_2_O_2_ and LT% were observed in Chardonnay (*P* < 0.0001) and Shiraz (*P* = 0.0017), but this trend was opposite in Grenache (*P* = 0.0005) (Fig. 4). A strong negative correlation between GABA concentration and LT% was observed in Chardonnay (*P* < 0.0001), Shiraz (*P* < 0.0001), and Grenache berries (*P* < 0.0001) (Fig. 4).

**Figure 4:**
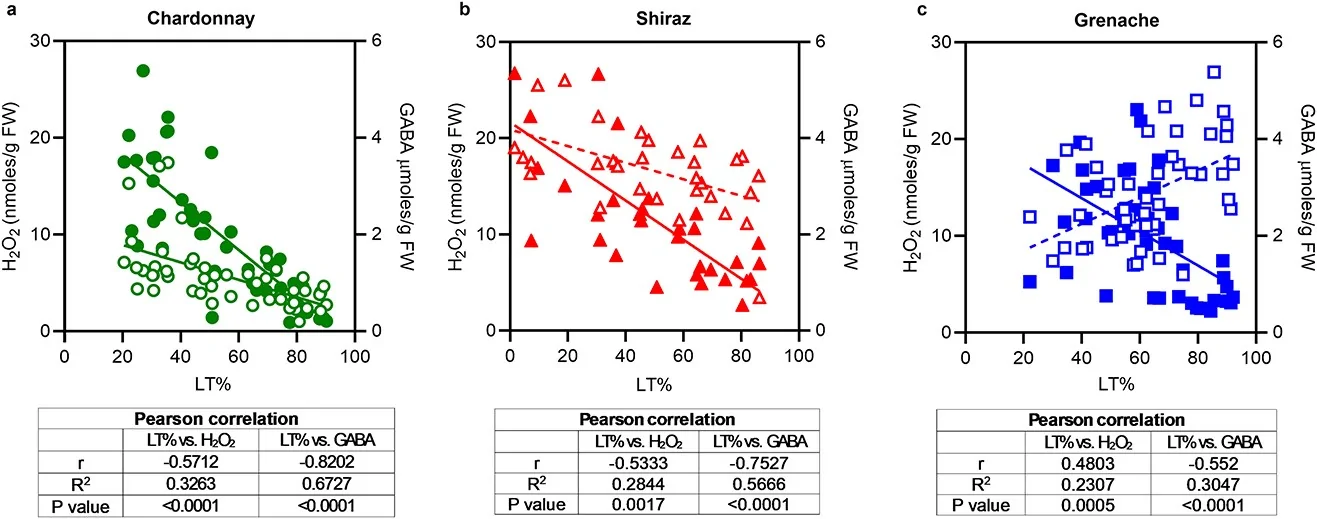
Pearson correlation between H_2_O_2_, GABA, and LT% of individual Chardonnay (a), Shiraz (b), and Grenache (c) berries. Empty symbols and dashed lines represent H_2_O_2_ while filled symbols and solid lines represent GABA. Data points combine four biological replicates of each season 1 (2018–19) and season 2 (2019–20).

APX activity significantly increased from the pea-stage until harvest in all three grape cultivars and then decreased at the postharvest stage (Fig. 5a and b; *P* < 0.0001). During season 1, there was a significant difference between Grenache and Chardonnay observed at postharvest, but during season 2, no significant differences between the cultivars were observed. CAT enzyme activity was low at the pea-stage and increased until postveraison (Fig. 5c and d). A separation between the CAT activity levels of the three cultivars was observed at postharvest, where Grenache berries had a greater activity level in both seasons (51.5; 47.1 Ug^−1^ FW) when compared to Chardonnay (28.4; 25.9 Ug^−1^ FW) or Shiraz (21.6; 30.7 Ug^−1^ FW) (Fig. 5c and d). Overall, Chardonnay and Shiraz followed similar APX and CAT activity trends during berry ripening, whereas Grenache berries maintained a greater level of CAT activity for a longer period (Fig. 5). Both the cultivar and developmental effects on CAT activity were found to be significant (*P* < 0.0001; Fig. 5).

**Figure 5:**
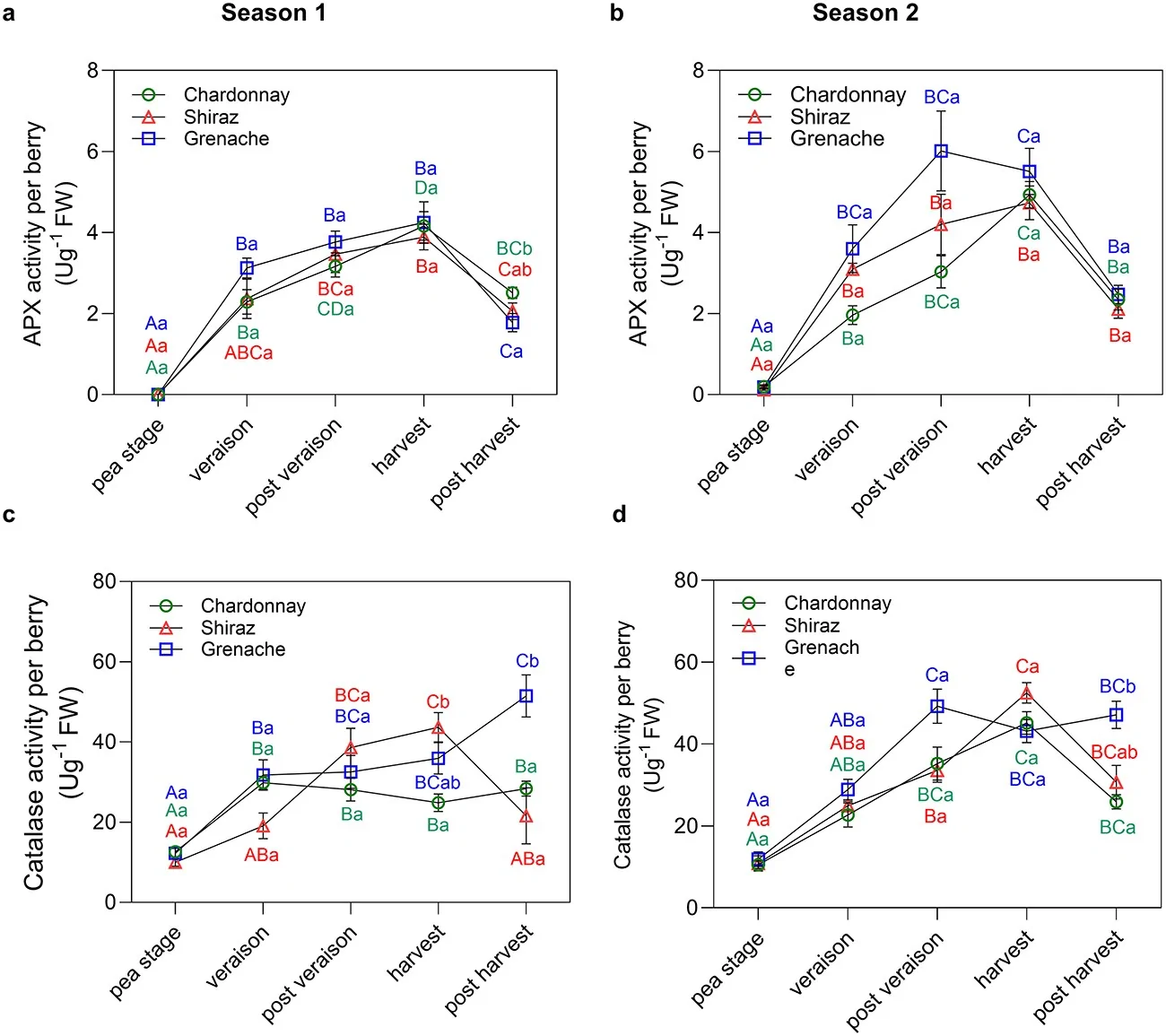
Changes APX and CAT activity in berries of Chardonnay (green circle), Shiraz (red triangle), and Grenache (blue square) berry development during seasons 1 and 2. Data are represented as ±SEM, *n* = 4. Uppercase letters describe significance within a cultivar across development while lowercase letters describe significance between cultivars at a singular developmental stage according to Tukeys’ multiple comparison (two-way ANOVA repeated measures, *P* < 0.05).

### Ripening can be delayed through exogenous GABA application

Grape berries were treated with dH_2_O (control) or 5 mM GABA weekly from pea-stage until the control berries reached veraison (defined as TSS of ~10–13 °Brix) during seasons 1 and 2. Phenotypically, the berries of all three grape cultivars treated with exogenous GABA spray appeared to ripen later than those treated with dH_2_O (Fig. 6). This observation is supported by quantitative physical measurements of the berries at veraison (Fig. 7, Supplementary Table S1). For both seasons, the GABA-treated berries had lower berry mass compared to control berries which had reached veraison in all three cultivars and this difference was significant for Chardonnay and Grenache in both seasons, but only significant during season 2 for Shiraz (Fig. 7a–c). GABA-treated berries displayed a lower TSS than the control; this was significant in both seasons for Shiraz and Grenache, but not significant during season 2 for Chardonnay (Fig. 7d–f). A significant increase in LT% for the GABA-treated berries was observed in all three grape cultivars across both seasons (Fig. 7g–i). The exogenous application of GABA to developing berries did not appear to result in an increase in intracellular GABA content, with no significant difference observed in any cultivar in either season (Fig. 8a–c, Supplementary Table S1). This treatment did however decrease berry H_2_O_2_ concentrations at veraison. This trend was observed for all cultivars in both seasons (Fig. 8d–f, Supplementary Table S1). In season 1, this difference was significant between control and GABA treatment for Shiraz and Grenache, and in season 2, for all three cultivars. The activities of APX (Fig. 8g–I, Supplementary Table S1) and CAT (Fig. 8j–l) increased with GABA treatment, but this observation was most apparent in season 2 for APX activity, where all three grape cultivars displayed a significant increase in APX activity. Furthermore, the application of GABA to the berries from pea-stage until veraison showed higher LT% as seen in the fluorescein diacetate (FDA)-stained images in Fig. 9a. Correlation analysis between LT% and TSS in GABA treated Chardonnay, Shiraz, and Grenache berries shows higher LT% and lower TSS compared to the controls (Fig. 9b).

**Figure 6:**
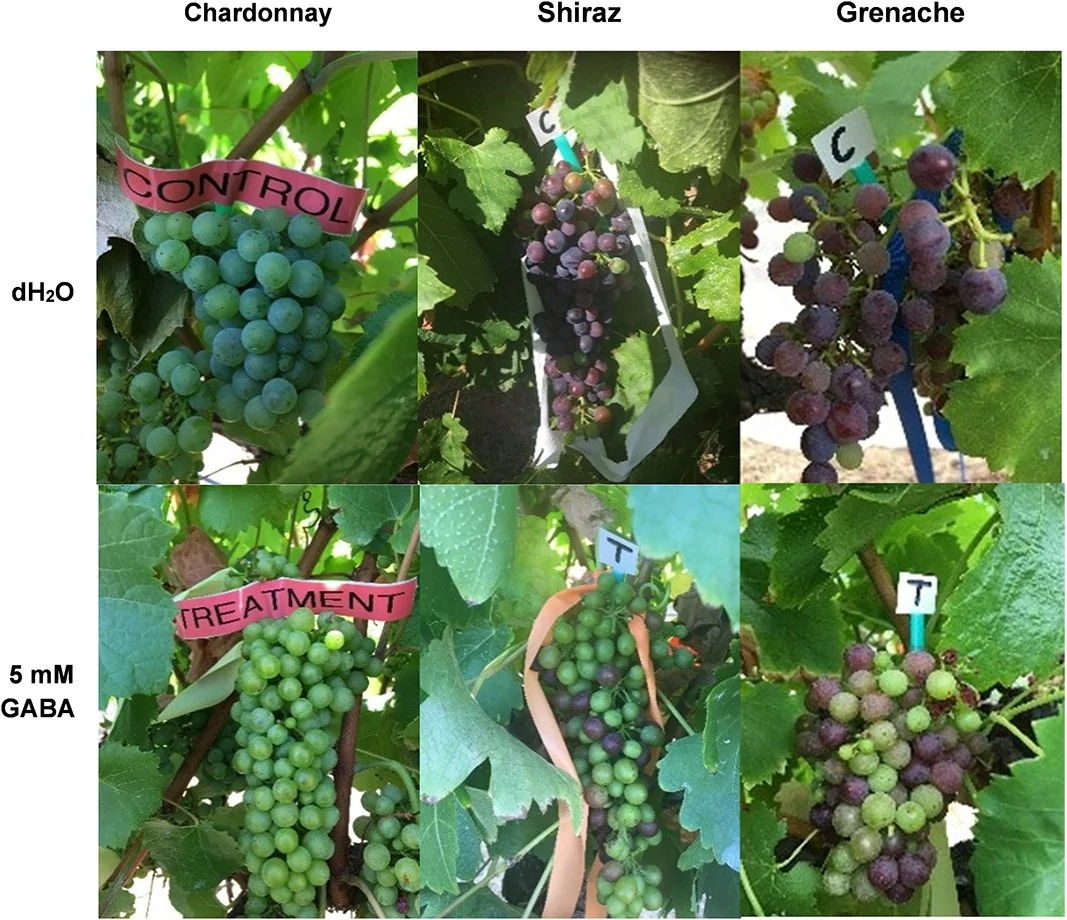
Representative images of control and GABA treated Chardonnay, Shiraz, and Grenache bunches when control berries reached veraison (°Brix between 10 and 12). Grape berries were treated with dH_2_O (control) or 5 mM GABA weekly from pea-stage until veraison during seasons 1 and 2. Grape berries in a bunch ripen asynchronously.

**Figure 7:**
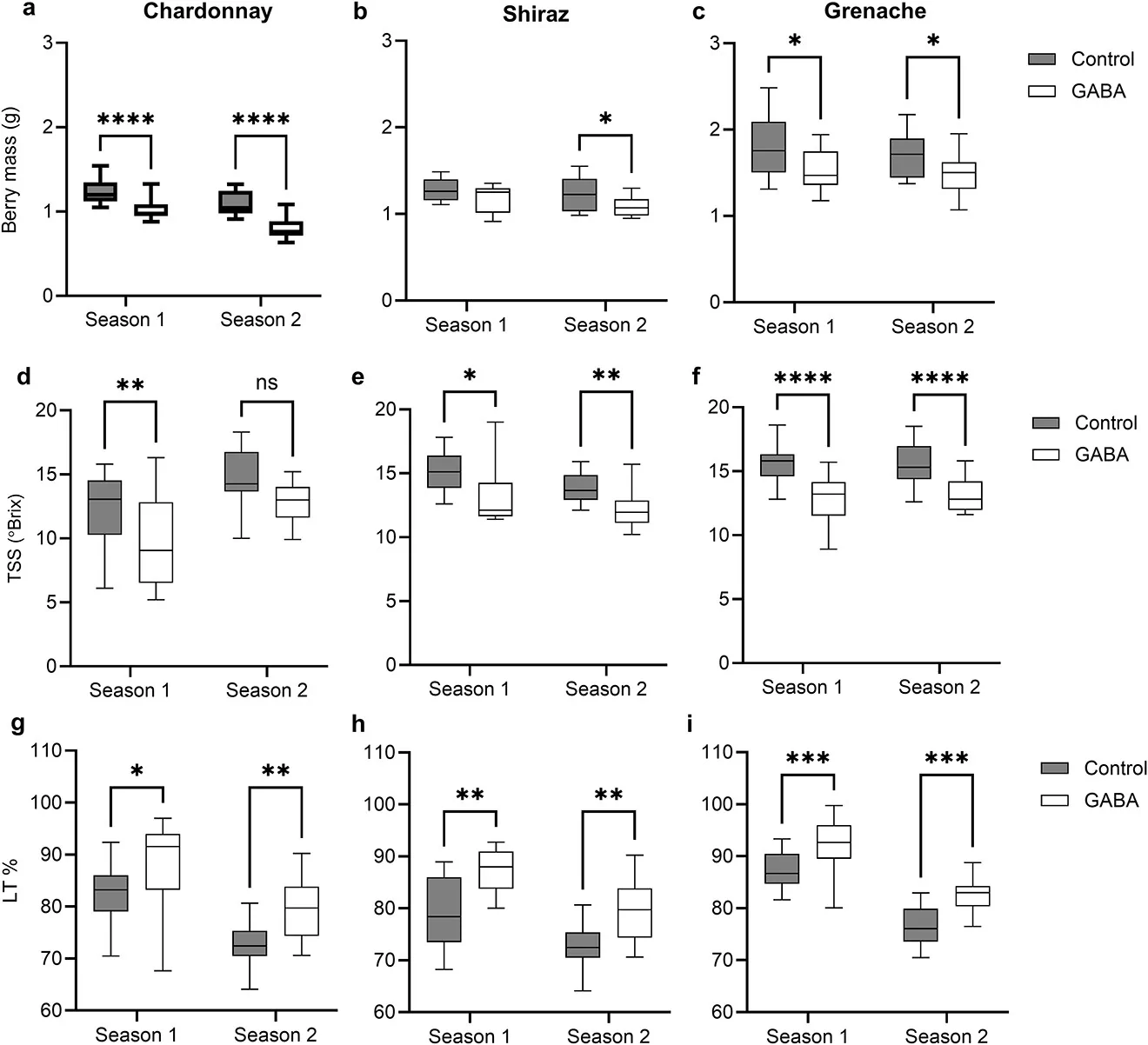
Physical changes in Chardonnay, Shiraz, and Grenache berries at veraison of control and GABA treated bunches. Grape berries were treated with dH_2_O (control) or 5 mM GABA weekly from pea-stage until veraison during seasons 1 and 2. FW (a–c), TSS (d–f), and LT% (g–h) was recorded for the three grape cultivar at veraison. Data are min to max box and whiskers plot of four biological replicates (*n* = 4), with significance between control and GABA-treated indicated according to Šídák's multiple comparisons test (^*^P < 0.05; ^**^P < 0.01; ^***^P < 0.001; ^****^P < 0.0001).

**Figure 8:**
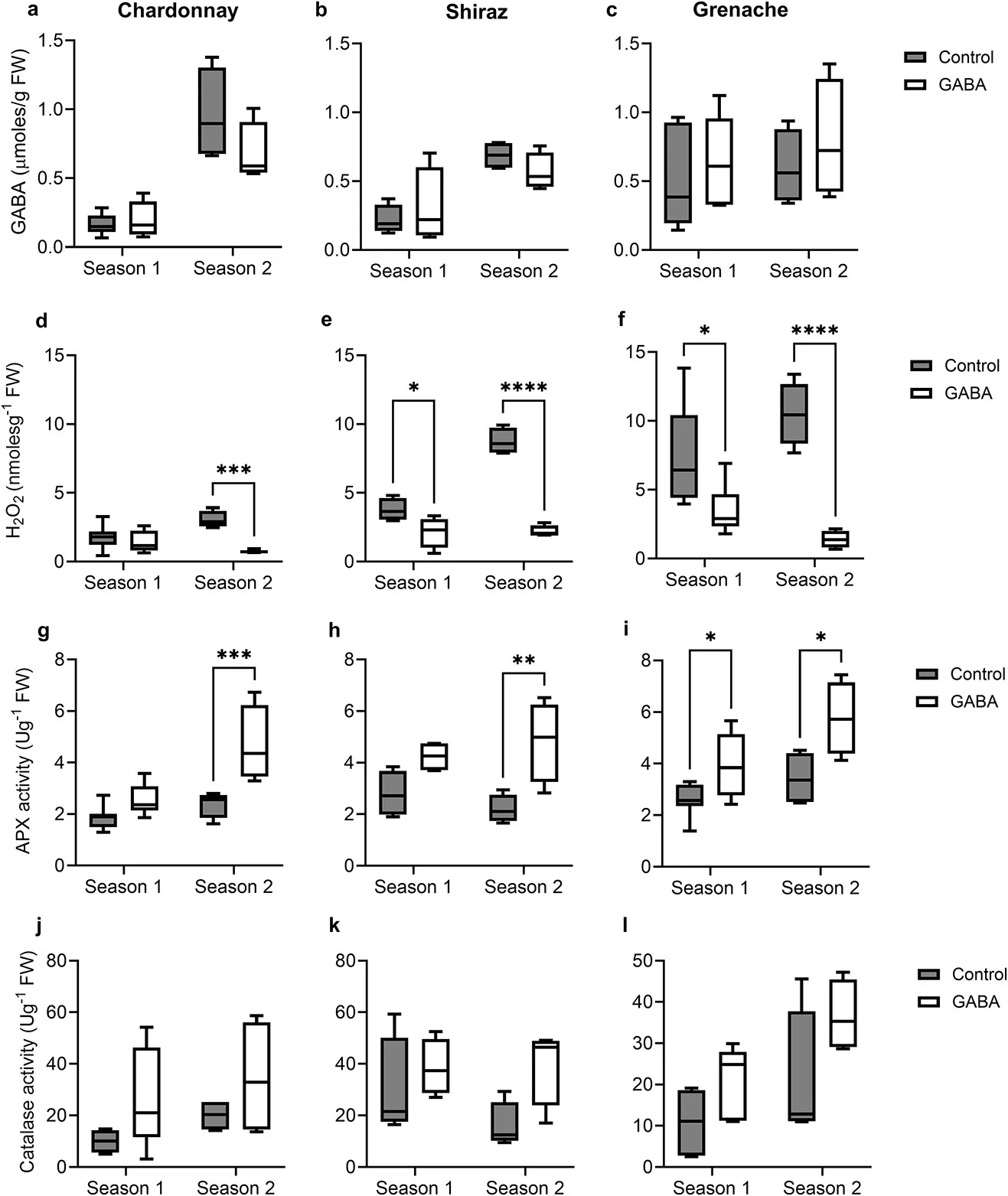
Biochemical changes in Chardonnay, Shiraz, and Grenache berries treated with GABA. Grape bunches were treated with dH_2_O (control) or 5 mM GABA weekly from pea-stage until veraison during seasons 1 and 2. GABA content (a–c), H_2_O_2_ content (d–f), APX activity (g–h), and CAT activity (j–l) was determined in the three-grape cultivar at veraison. Data are min-to-max box and whiskers plot of four biological replicates (*n* = 4), with significance between control and GABA-treated indicated according to Šídák's multiple comparisons test (^*^P < 0.05; ^**^P < 0.01; ^***^P < 0.001; ^****^P < 0.0001).

**Figure 9:**
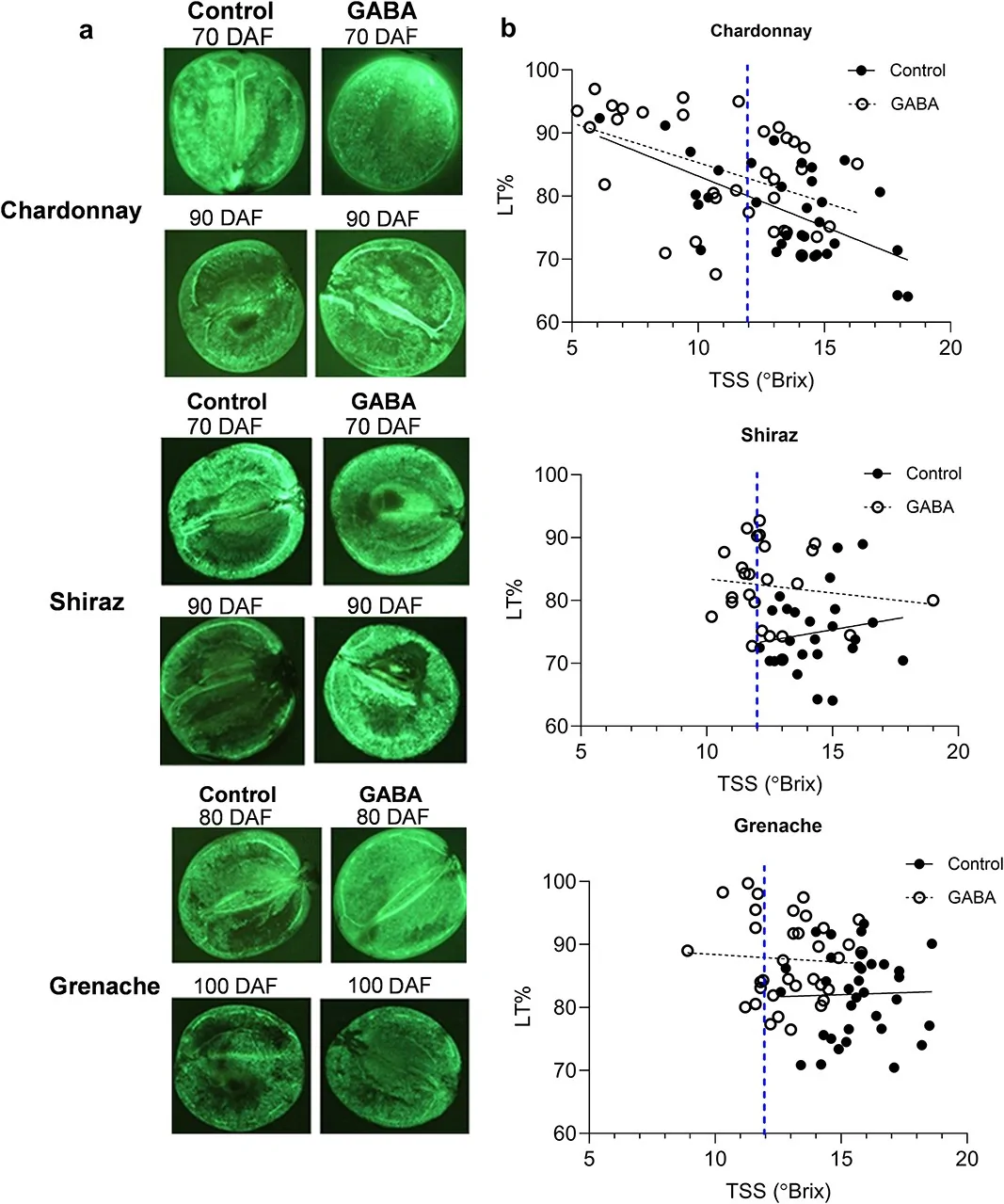
Effect of GABA application on LT% in Chardonnay, Shiraz, and Grenache berries. Grape bunches were treated with dH_2_O (control) or 5 mM GABA weekly from pea-stage until veraison during seasons 1 and 2. (a) LT% in representative images of all berries sampled when controls reached veraison and postveraison (70 and 90 DAF, for Chardonnay and Shiraz; 80 and 90 DAF for Grenache). Green fluorescence indicates living tissue while black region indicates cell death. GABA-treated berries stained with FDA show higher LT% (green fluorescence) compared to controls sampled at veraison and postveraison stages. (b) Correlation analysis between LT% and TSS in Chardonnay, Shiraz, and Grenache shows higher LT% and lower TSS compared to the controls. Each data point represents one berry. The dotted vertical line represents the TSS (°Brix between 10 and 12) at which berries enter veraison. GABA treated berries lie predominantly to the left of the dotted line, i.e. TSS < 12 suggesting delayed onset of ripening.

## Discussion

### Distinct differences in ripening of Chardonnay, Shiraz, and Grenache berries

Veraison marks the onset of ripening, leading to numerous changes in the chemical composition and berry physiology (Coombe 1992). The berry chemical composition is altered through significant changes in metabolism and results in a doubling of size, decline in acidity, and increase in softness, colour, and flavour development (Robinson and Davies 2000, Pereira et al. 2006). Cultivar-dependent variation in berry ripening has been well documented (Sweetman et al. 2009, Shiraishi et al. 2010, Sadras and Petrie 2012, Kuhn et al. 2013, Río Segade et al. 2013, Griesser et al. 2024). In the present study, Chardonnay, Shiraz, and Grenache berries varied in their mass and LT% (Fig. 1). Grenache berries tended to be larger in mass, and this was accompanied by a greater LT%, whereas Chardonnay and Shiraz followed more similar trends. In season 1, an increase in berry mass was observed from veraison to harvest, whereas in season 2, this increase was only from veraison to postveraison (Fig. 1); this may be explained by the harsher climatic conditions in the second growing season (Table 1). The observed variability in weather between the two growing seasons is natural, but can be used to understand metabolic patterns during the growth and maturation of grape berries (Serrano-Megías et al. 2006; Wissemann and Lee 1980).

The steep decrease in berry mass observed for both Chardonnay and Shiraz, but not Grenache, is most likely due to water loss in late ripening, also known as ‘berry shrivel’. Berry shrivel results from a combination of transpiration and xylem back-flow processes and is cultivar-dependent (Tilbrook and Tyerman 2008). Shiraz is particularly prone to berry shrivel, accelerated by hot and dry growing conditions, and occurs when the berries reach maximum weight, leading to yield losses of up to 30% (McCarthy and Coombe 1999, Rogiers and Holzapfel 2015). A recent study of normal versus shrivelled berries sampled from the same Shiraz cluster showed an overall loss in fresh berry mass as the TSS increases in shrivelled berries whilst normal berries had higher fresh mass and lower TSS (Deloire et al. 2021). Our data do not indicate a great level of variability in the TSS amongst the three cultivars at a given timepoint (Fig. 1), but this is not surprising as ripening is coordinated within each berry on a bunch (Coombe 1992). Asynchronous ripening of individual berries is better explored in Fig. 2, where the TSS and LT% of individual berries sampled from within and across berry bunches are plotted together. The majority of variability in TSS between individual berries can be explained by LT%, which is typical during the grape ripening period (Pirie and Mullins 1977, Sweetman et al. 2009).

In both seasons, significant decrease in LT% from postveraison to postharvest was observed in Chardonnay and Shiraz berries, with Shiraz showing the lowest LT% at postharvest (Fig. 1e and f), consistent with Bonada et al. (2013) and Xiao et al. (2018). A higher LT% was observed in Grenache at harvest compared to the other two cultivars. This observation may be explained by the isohydric and anisohydric differences between the three cultivars, with Shiraz and Chardonnay being generally regarded as anisohydric while Grenache is isohydric (Schultz 1995, Schultz 2003, Rogiers et al. 2009). Grenache berry clusters that display isohydric behaviours are less prone to berry shrivel than Shiraz due to maintained hydraulic resistance during ripening (Scharwies and Tyerman 2017). Our data suggest that anisohydric Chardonnay and Shiraz berries ripened quicker than those of isohydric Grenache berries as reflected by increased TSS and EtOH production, and decreased LT% and berry mass (Figs. 1−3). These observations fit with (Fuentes et al. 2010), who suggest that berry cell death is a cultivar-dependent phenomenon in a study on berries from 22 cultivars. Furthermore, berries from bunches with TSS greater than 24 °Brix, showed a steady decline in cell vitality around the vasculature, with the highest cell death near the seeds (Clarke et al. 2010, Hoff et al. 2021), consistent with our FDA-stained berry images (Fig. 2).

### Metabolic shifts in berry development

During berry ripening, hypoxia is induced in the berry, partly due to reduced oxygen diffusion through changes in the berry skin and decreased porosity in the berry mesocarp, likely resulting in decreased membrane integrity and ultimately cell death (Blokhina et al. 2001, Tilbrook and Tyerman 2008, Xiao et al. 2018). Under hypoxic conditions the energy requirements of the cell are met through fermentation, resulting in EtOH production, and contributing to unregulated ROS accumulation (Blokhina et al. 2001, Cukrov et al. 2016). EtOH concentrations steadily increased during ripening for all three varieties, but this effect was less pronounced in Grenache berries and most prevalent in Shiraz berries, suggesting varietal differences in hypoxia-induced cell death. These varietal differences in the H_2_O_2_ concentrations are largely explained by LT% for Shiraz and Chardonnay, but not Grenache, which may be associated with higher CAT activity (Fig. 4). This may be a result of the high LT% of Grenache berries at later sampling points. Interestingly TSS was similar between the three cultivars across both seasons regardless of LT% (Fig. 2). At 50% LT, Grenache berries displayed the highest average TSS of 26.2 ^°^Brix, while Chardonnay and Shiraz berries were more similar with 21.4 and 20.6 ^°^Brix, respectively. These observations reveal distinct cultivar-dependent patterns in cell death and TSS accumulation.

The metabolic function of GABA accumulation during ripening is still unclear and, in this study, little varietal variability was observed at any given sampling point (Fig. 3). Biais et al. (2010) suggest that stress, such as hypoxic pockets which form during melon ripening, can trigger GABA accumulation. This may also occur in the grape berry. This accumulation may protect against oxidative damage during development or adverse growing conditions (Aghdam and Fard 2017, Salah et al. 2019). When blueberries were treated with 1 mmol/L GABA; superoxide dismutase, CAT, glutathione reductase, ascorbate peroxidase, and peroxidase enzyme activities increased, while H_2_O_2_ content decreased, indicating that GABA has a role in delaying senescence postharvest (Ge et al. 2018). Previous studies have also implicated a protective role of GABA through promoting antioxidant capacity and ROS detoxification under stress (Vergara et al. 2012, Abd El-Gawad et al. 2021, Bhardwaj et al. 2021, Ren et al. 2021). During both growing seasons, we observed that the activity of antioxidant enzymes, APX, and CAT, were similar between the three cultivars until harvest, but CAT activity was notably higher in Grenache berries postharvest (Fig. 5). The maintained CAT activity in Grenache berries may help explain the decrease in H_2_O_2_ from veraison to postharvest and greater LT% compared to the other two cultivars.

### Onset of veraison in Chardonnay, Shiraz, and Grenache berries is delayed by GABA application

Changing climate with increasing frequency of warmer temperatures during growing seasons often results in grapes ripening earlier and impacts yield and berry quality (Palliotti et al. 2014). Wine produced from such berries typically result in higher alcohol content with less than optimal flavour, aroma, and colour (Sadras and Moran 2012, Poni et al. 2018). Vineyard management techniques, such as vine shading (Tiffon-Terrade et al. 2023), pruning (Martínez de Toda and Balda 2015, Martinez de Toda 2020), crop load and irrigation (Previtali et al. 2021, Previtali et al. 2022a, Green et al. 2024), or the application of plant hormones, such as auxin (Davies et al. 1997, Böttcher et al. 2012, Dal Santo et al. 2020, Davies et al. 2022) or ethylene (Chervin et al. 2004, Ttcher et al. 2013, De Santis et al. 2021), are being trialled to manage fruit ripening and harvest.

Strategies to mitigate abiotic stresses, such as drought, heat, salinity, and cold, have demonstrated the efficacy of exogenous GABA treatment either as foliar spray or as soil drenching to be effective in crops (Hu et al. 2015, Priya et al. 2019, Abd El-Gawad et al. 2021, Ren et al. 2021, Aljuaid and Ashour 2022, Hayat et al. 2023). These exogenous applications appear to reduce ROS levels, maintain membrane integrity, and osmolarity within the cells, induce antioxidant enzymes, and lead to crosstalk with other phytohormones. GABA pretreatment has proved effective in postharvest storage applications, preserving fruit quality, and preventing chilling injuries (Wang et al. 2014, Li et al. 2019, Asgarian et al. 2022, Zhou et al. 2022). The literature currently does not describe the role of GABA spray on grape clusters in delaying the onset of veraison, but there is a market requirement for this not only in viticulture but also in other industries, such as avoiding forage crops developing grain. Our findings suggest that weekly foliar application of 5 mM GABA to berry bunches can delay the onset of veraison (Fig. 6), reducing berry mass and TSS while increasing LT% (Fig. 7). This trend was observed in all three investigated cultivars, but the effect was most pronounced in Chardonnay and Grenache.

Exogenous treatment did not appear to increase intracellular GABA concentrations in the grape berry, but did decrease H_2_O_2_ accumulation at veraison, suggesting a potential role in modulating oxidative environments linked to cell vitality. GABA has been approved as food grade up to 100 mg/serving in the USA with no serious adverse events reported in studies of 120 mg/day for a period of 12 weeks (Yamakoshi 2006, Oketch-Rabah et al. 2021). Our data have demonstrated that there is an increase in activity of the antioxidant enzymes APX and CAT until harvest (Fig. 5), likely in response to hypoxia stress induced from increasing fermentation during berry ripening. Exogenous GABA application was associated with increased APX activity in GABA treated berries, facilitating H_2_O_2_ scavenging through the ascorbate-glutathione cycle. The conversion of H_2_O_2_ to water will help to reduce the accumulation of ROS which when unregulated could result in cell death through lipid peroxidation leading to damage to other cellular structures. While non-significant, GABA application was also associated with an increase in the activity of CAT, another important ROS scavenging enzyme. These positive effects observed in antioxidant capacity are typical of GABA pretreatment observed in other studies (Wang et al. 2014, Ge et al. 2018, Li et al. 2019, Asgarian et al. 2022).

## Conclusion

The berries of three winegrape cultivars showed differences in the concentrations of ROS, GABA, and antioxidants across the two seasons in this study, with Grenache having a higher LT% at harvest, probably associated with low hypoxia (low EtOH), H_2_O_2_ accumulation, and high CAT activity. There was no correlation between LT%, ROS, and GABA concentrations in Grenache when compared to the other two cultivars. Exogenous application of GABA to the berries from pea-stage delayed veraison with significant decrease in ROS, berry mass and TSS at veraison across both seasons. One interpretation is that the application of exogenous GABA may have led to the suppression of growth. However, treated berries had lower ROS (spike in ROS triggers veraison) levels and higher APX (breakdown of ROS to water) levels suggesting that the effect was most likely due to reduced ROS levels that triggered a delay in the onset of ripening and not to growth suppression. There is a need for further research to include transcriptomic and signalling studies to understand the mechanistic action of exogenous GABA application. Extended studies of berries treated with GABA from pea-stage to harvest would provide insights into the length and duration this treatment can delay veraison and potentially improve the LT% and berry quality particularly in cultivars, such as Shiraz. Exogenous application of GABA can be used to delay the initiation of ripening to mitigate adverse weather events and potentially improve the quality of berries prone to more cell death, such as Shiraz.

## Materials and Methods

### Sampling and treatment design

Own-rooted Shiraz (clone BVCR 12) and Chardonnay (cl. I10V1) vines planted in 1993 from Coombe vineyard (34°58′03.12″ S and 138°38′00.21″ E), and Grenache vines on own roots from Alverstoke vineyard, (34°96′82.8″ S and 138°63′74.2″ E) at the Waite Campus of The University of Adelaide were chosen for this study. The vines were grown under standard vineyard management with vertical shoot positioning, spur pruned (two buds), drip irrigation on dark brown clay soils with shale fragments, grading into red-brown mottled clay, and with overlying olive-brown mottled cracking clay. Vines were trained to a vertical shoot positioned trellis with north–south row orientation and vine and row spacing of 2.7 by 3.0 m, respectively.

Sampling was carried out over two seasons, 2018–19 (season 1) and 2019–20 (season 2), from the pea-stage (30 DAF) for all the cultivars. For Shiraz and Chardonnay sampling occurred at the veraison stage (70 DAF; 10–12 °Brix), postveraison (90 DAF), harvest (110 DAF), and postharvest stages (120 DAF; > 25 °Brix). For Grenache, sampling was carried out at veraison (80 DAF), postveraison (100 DAF), harvest (118 DAF), and postharvest (128 DAF) stages. Veraison dates for the three cultivars for both seasons are shown in Table 2.

**Table 2:** Dates for veraison for Chardonnay, Grenache, and Shiraz in seasons 1 and 2.

	Chardonnay	Grenache	Shiraz
**Season 1 − Veraison**	70 DAF (05 January 2019)	79 DAF (19 January 2019)	68 DAF (12 January 2019)
**Season 2 − Veraison**	72 DAF (11 January 2020)	77 DAF (16 January 2020)	70 DAF (12 January 2020)

The experimental design consisted of berries harvested from bunches on vines from four different biological replicates (block); each biological replicate had four vines (Shiraz) or six vines (Chardonnay and Grenache). A total of 10 bunches per biological replicate were tagged for sampling (4 biological replicates × 10 bunches per replicate = 40 bunches in total). Three berries per bunch were sampled at each stage with a total of 30 berries per biological replicate. From each replicate, four berries were used for fresh measurements and remaining berries frozen for biochemical assays.

For exogenous GABA application experiments, the experimental design was similar to the above except, a total of 20 bunches per biological replicate were tagged for sampling with 10 bunches for control and 10 bunches for GABA treatment. The tagged bunches were sprayed weekly with either distilled water containing 0.03% (v/v) Silwet-77 (control) or 5 mM GABA solution containing 0.03% (v/v) Silwet-77 (Plant media, OH, USA) during both seasons until control berries reached veraison (defined as °Brix between 10 and 12). Three berries per bunch per treatment were sampled at veraison with a total of 30 berries per biological replicate. From each replicate, four berries were used for fresh measurements and remaining berries frozen for biochemical assays.

Climatic parameters, such as ambient temperature, VPD, and solar radiation, were measured by an Measurement Engineering Australia (MEA) weather station in the Alverstoke vineyard from the Waite campus. Data of the monthly average temperature, monthly maximum VPD, and mean daily solar radiation registered at the experimental vineyard are shown in Table 1.

### Physical grape berry measurements

The mass (g) of the individual berries was recorded at each sampling stage. The berry was cut into two halves. One-half of the berry was crushed for the juice to measure TSS with a digital refractometer (Atago, Tokyo, Japan). The other half was placed into one well of a 12-well transparent polystyrene plate (sterilized with lid, flat bottom; Corning Costar, Sigma) to measure cell vitality using FDA according to (Tilbrook and Tyerman 2008). Briefly, the FDA dye solution and an appropriate sucrose concentration (calculated based on berry osmolarity) was applied to the berry half and incubated for 30 min in the dark. FDA-stained berries were viewed under a Nikon SMZ 800 (Nikon Co., Tokyo, Japan) dissecting microscope under ultraviolet light at minimal magnification (0.5× objective lens). Images were obtained with the same gain and exposure settings using a Nikon DS-5Mc colour-cooled digital camera (Tochigi Nikon Precision Co., Ltd, Otawara, Japan) and NIS-Elements F2.30 software (Tilbrook and Tyerman 2008). The images were processed using the image processing code according to (Fuentes et al. 2010) within MATLAB R2019a software (Mathworks, Natick, MA, USA) to quantify the cell vitality (LT%).

### Quantification of GABA in grape berries

GABA quantification was carried out following a modified protocol (Zhang and Bown 1997, Ramesh et al. 2015). Briefly, Glutamate Decarboxylase (GAD) was inactivated by the addition of 400 μL methanol to ~100 mg of frozen powdered grape berry samples and incubation at 25°C for 10 min. The samples were vacuum dried and resuspended in 1.0 mL of 70 mM LaCl_3_. The tubes were kept in a shaking incubator at 100 revolutions per minute for 15 min and centrifuged at 9500 *g* for 5 min. The supernatant was collected, and lanthanum was precipitated by centrifugation in the presence of 1 M KOH. The resulting supernatant was used to quantify GABA using the GABase enzyme (Sigma-Aldrich, Macquarie Park NSW) by measuring reduction of NADP^+^ to NADPH at 340 nm using a FLUOstar Omega plate reader (BMG Labtech, Ortenberg, Germany).

### Quantification of H_2_O_2_

H_2_O_2_ was extracted from frozen berry tissue following (Pilati et al. 2007) and quantified by a fluorimetric assay using the Amplex Red Hydrogen Peroxide/Peroxidase Assay (Thermo Fisher, Waltham, MA, USA). A total of 200 mg of finely ground berry power was solubilized in 500 μL of sodium phosphate buffer (50 mM, pH 7.4) on ice for 5 min. Following centrifugation at 14 000 *g* for 20 min, an equal volume of the cleared supernatant was added to a 2:1 (v/v) chloroform: methanol, centrifuged at 8000 *g* for 5 min and 50 μL of the upper aqueous phase was transferred into a black 96-well microplate (clear bottom, Elisa plate; Greiner Bio-One, Australia). The working solution from Amplex Red Hydrogen Peroxide/Peroxidase Assay (Thermo Fisher, Waltham, MA, USA) was added to the extract and incubated in the dark for 30 min at 25°C. The fluorescence was measured using a FLUOstar Omega plate reader (BMG Labtech, Ortenberg, Germany) at 540/590 nm and relative quantification of H_2_O_2_ from the samples was performed by comparison to a H_2_O_2_ standard curve.

### Quantification of CAT and APX enzyme activity

CAT was extracted from 500 mg of frozen berry powder in 2 mL of 50 mM potassium phosphate buffer (pH 7.0) containing 2 mM EDTA and 1% (w/v) polyvinylpyrrolidone at 4°C. The extraction mixture was centrifuged at 10 000 *g* for 10 min at 4°C and the supernatant was collected for CAT enzyme activity assay (Paciello et al. 2017). CAT enzyme measurements were carried out using the CAT Assay Kit (Megazyme International Ireland Ltd, Wicklow, Ireland) following the manufacturer's instructions.

APX extraction and activity assay were carried out as mentioned in Sun et al. (2011) with slight modifications. APX was extracted from 500 mg of the frozen berry samples using 2 mL of 0.1 M sodium phosphate buffer (pH 7.0) for 10 min at 4°C and cleared by centrifugation at 12 000 *g* for 15 min. The resulting supernatant (10 μL) was combined with 290 μL of reaction mixture containing 0.5 mM of ascorbic acid, 0.1 mM of EDTA-Na_2_, and 0.1 of mM H_2_O_2_. Oxidation of ascorbic acid was measured as a decrease in absorbance at 290 nm using a FLUOstar Omega plate reader (BMG Labtech, Ortenberg, Germany). One APX enzyme unit was defined as 1 μmole of ascorbic acid oxidized to mono dehydroascorbic acid by APX per minute at 290 nm (Sun et al. 2011).

### Quantification of EtOH

EtOH was quantified using an EtOH Assay kit following the manufacturer's instructions (Megazyme International Ireland Ltd, Wicklow, Ireland). Approximately 2 g of the frozen berry powder was allowed to thaw at room temperature. The sample was centrifuged at 9500 *g* for 15 min (Xiao et al. 2018). An aliquot of 10 μL of supernatant was used to quantify EtOH at 340 nm using a FLUOstar Omega plate reader (BMG Labtech, Ortenberg, Germany). EtOH is quantified based on the principle that in the presence of alcohol dehydrogenase, EtOH reacts with NAD^+^ to form acetaldehyde and NADH. This reaction is trapped by the presence of aldehyde dehydrogenase, which converts acetaldehyde to acetic acid. The absorbance of NADH at 340 nm corresponds to the quantity of EtOH present in the sample.

### Statistical analysis

All statistical analyses were performed using GraphPad prism 10.2 software (San Diego, CA, USA). Differences between samples were examined for significance using a two-way analysis of variance (ANOVA) repeated measures with Tukey's multiple comparison test. Differences at the *P* < 0.05 level were considered significant. Pearsons’s correlation analysis was used for assessing linear relationships using a two-tailed test.

## Supplementary Material

Supplementary_table_S1

## Data Availability

The authors confirm that the data supporting the findings of this study are available within the article [and/or] its supplementary materials.
